# Introduction of monkeypox virus in Benin, 2022

**DOI:** 10.1186/s40779-022-00424-w

**Published:** 2022-11-09

**Authors:** Anges Yadouleton, Martin Faye, Carine Tchibozo, Mariam Oke, Oumar Faye, Thierry Lawale, Eric Denon, René Keke, Ange Dossou, Alban Zohoun, Francis Dossou, Moussa Moise Diagne, Sourakou Salifou, Khadija Leila Diatta, Mignane Ndiaye, Safietou Sankhe, Amadou Diallo, Sonia Bedie, Clément Glele-Kakai, Al Fattah Onifade, Raoul Saizonou, Gildas Hounkanrin, Olga Quenum, Yvette Badou, Benjamin Hounkpatin, Amadou Alpha Sall, Ousmane Faye

**Affiliations:** 1 Laboratoire des Fièvres Hémorragiques Virales (LFHV), Ministry of Health of Benin, 01-882 Cotonou, Benin; 2 Ecole Normale Supérieure de Natitingou, Université Nationale des Sciences, Technologies, Ingénierie et Mathématiques (UNSTIM), 2282, Goho, Abomey, Cotonou, Benin; 3 Virology Department, Institut Pasteur de Dakar, 36 Avenue Pasteur, 220, Dakar, Senegal; 4 Ministry of Health of Benin, 01-882 Cotonou, Benin; 5 National Public Health Laboratory, Ministry of Health of Benin, 01-882 Cotonou, Benin; 6 Epidemiology, Clinical Research and Data Science Department, Institut Pasteur de Dakar, 220, Dakar, Senegal; 7 WHO Country Office in Benin, Avenue CENSAD - Les Cocotiers, 01, BP 918, Cotonou, Benin

**Keywords:** Monkeypox virus, Introduction, Human-to-human transmission, Benin

Dear Editor,

Monkeypox is an infectious disease that is endemic in a dozen of African countries. Some imported cases have been also reported outside of Africa in the past [1]. Since early May 2022, monkeypox infections including human-to-human transmission, were reported in a multi-country outbreak in non-endemic countries and declared Public Health Emergency of International Concern (PHEIC) by the World Health Organization (WHO) in July 2022 [2]. As of 20 September 2022, a total of at least 62,798 human cases of monkeypox with 20 deaths have been confirmed in 115 countries in five WHO regions [3].

Typically, the clinical hallmark of monkeypox disease includes fever, rash and swollen lymph nodes, and Monkeypox virus (MPXV) is transmitted to humans through close contact with an infected person or animal. MPXV is currently classified into two lineages, the West and Central African clade. However, a novel classification into MPXV clades 1, 2 and 3 has recently been proposed for the prior Central African clade, the prior West African clade and most of the MPXV strains characterized in human outbreaks from 2017, 2018 and 2022 [4].

Herein, we describe the first cases of monkeypox infections detected in Benin since 1978. On 1 June 2022, 3 out of 6 suspected monkeypox cases initially diagnosed positive at the Laboratoire des Fièvres Hémorragiques Virales (LFHV) in Benin were confirmed at the Institut Pasteur de Dakar in Senegal on 13 June 2022. The Benin National Ethical Committee at the Benin Ministry of Health (BMoH) approved the surveillance protocol as a less than minimal-risk research, and written consent forms were not required. Oral consent was obtained from all patients included in this study. All methods including the use of human samples were performed in accordance with the Declaration of Helsinki. Positive samples were sequenced using an enrichment library preparation method and sequences were analysed using phylogenetic analysis.

The three confirmed cases included a couple from the Ifangni district, Southern Benin (37-year-old man returning from Nigeria and 29-year-old woman) and an indigenous 15-year-old male child from the Bonou district (Southern Benin). The Cycle threshold (Ct) values at confirmation were 22.27, 23.44 and 34.00, respectively (Additional file 1: Table S1). All the patients were alive and not hospitalized. They presented no complications and had received no specific treatment. They were advised to practice self-isolation until the diagnosis confirmation was obtained.

Nearly complete genome sequences were obtained and sequences have been submitted to GenBank under the accession numbers OP422631, OP422632 and OP422633 for isolates BEN-22-MPOX 002, BEN-22-MPOX 003 and BEN-22-MPOX 004, respectively. The new MPXV sequences from Benin belonged to the same strain and clustered with 2021–2022 US sequences imported from Nigeria (ON676707, ON675438 and ON674051). All the sequences in this cluster originated from a sequence isolated from Nigeria in 2020 (MT903338) and are part of the prior Western African clade (recently proposed as MPXV clade 2) [4] (Fig. 1).

**Fig. 1:**
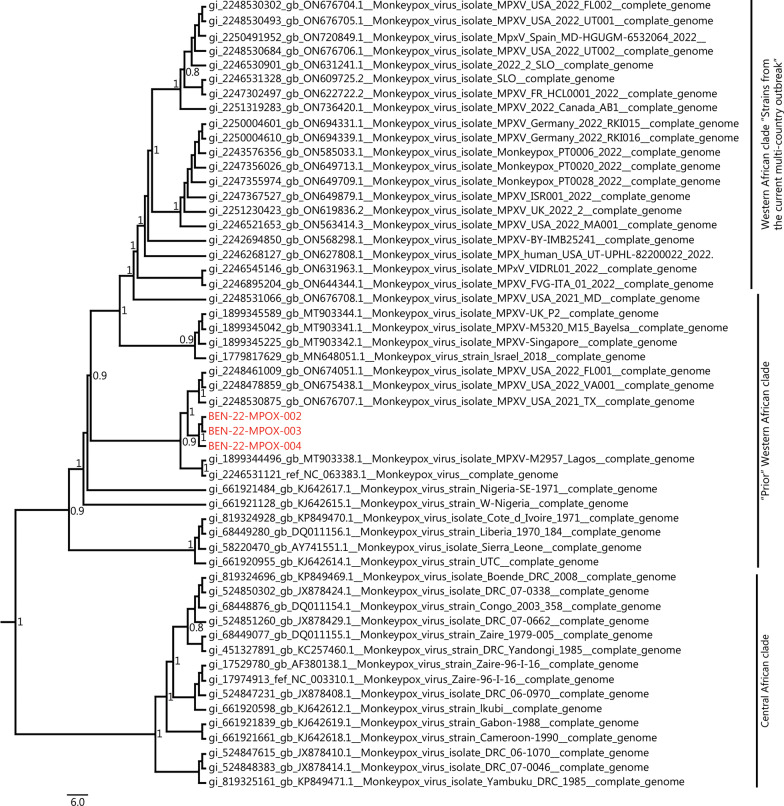
Maximum likelihood (ML) phylogenetic tree with 54 whole genome sequences, including 51 previously available in NCBI GenBank and representative of the current genetic diversity. The ML tree was inferred with the IQ-TREE web-server for 1000 ultra-fast bootstrap replicates under the K3Pu + F + I substitution model as estimated by ModelFinder and rooted to the earliest MPXV sequence (KJ642614). The topology was visualized by FigTree (v.1.4.2) (http://tree.bio.ed.ac.uk/software/figtree/). Only SH-like values ≥ 0.8 were shown on the tree. Genomes highlighted in red correspond to the newly characterized MPXV sequences from Benin, belonging to the prior Western African clade (proposed as MPXV clade 2) [4]

Benin is listed among the endemic African countries for monkeypox since an imported case had been previously documented on 22 November 1978 from Oyo State in Nigeria [5], where the MPXV is still circulating to date.

The introduction of MPXV from Nigeria which shares borders with Benin highlighted the potential for virus spread through direct or indirect population movements. Therefore, it is important to raise awareness among health professionals for the rapid identification and diagnosis of this disease in endemic countries such as Benin. In addition, WHO should make available diagnostics and vaccines worldwide, particularly to people in endemic countries in Africa where resources are limited, and reinforce the disease’s surveillance at local, regional and global levels. Many Western countries are also gearing up to start immunization campaigns. However, vaccine equity could be considered, targeting also populations and healthcare workers living in remote, forested areas in endemic countries in Africa, who encounter wildlife that carry monkeypox.

The current PHEIC could be probably a consequence of increased local transmission of MPXV in endemic countries in Africa such as Nigeria. Further clinical and genomic studies could be promoted for better understanding of the current epidemiology of monkeypox, as two new lineages have been recently identified in the US with a recurrent and dominant involvement of APOBEC3 activity in recent West African MPXV evolution.

## Supplementary Information

**Additional file 1: Table S1.** Description of suspected cases and PCR results.

## Data Availability

All data generated or analysed during this study are included in this published article. The newly characterized MPXV sequences from Benin have been deposited in GenBank under the accession number OP422631-33.

## Abbreviations

BMoH: Benin Ministry of Health
Ct: Cycle threshold
LFHV: Laboratoire des Fièvres Hémorragiques Virales
MPXV: Monkeypox virus
ML: Maximum likelihood
PHEIC: Public Health Emergency of International Concern
WHO: World Health Organization
