# Supplementary material for: Introduction of monkeypox virus in Benin, 2022
Source: Mil Med Res. 2022 Nov 9;9:63. doi: 10.1186/s40779-022-00424-w (PMC9644551; doi:10.1186/s40779-022-00424-w)
Supplement: Supplementary file 1 — Additional file 1: Table S1. Description of suspected cases and PCR results. [file 40779_2022_424_MOESM1_ESM.pdf]

Table S1 Description of suspected cases and PCR results

No.	Patient ID	Age (year)	Sex	Type of specimens	Date of sample collection	PCR results
1	BEN-22-MPOX 001	1	Female	Serum	June 1, 2022	Negative
2	BEN-22-MPOX 001	1	Female	Skin swab of the lesions	June 1, 2022	Negative
3	BEN-22-MPOX 002	37	Male	Serum	June 2, 2022	Negative
4	BEN-22-MPOX 002	37	Male	Skin swab of the lesions	June 2, 2022	Positive (Ct: 22.27)
5	BEN-22-MPOX 003	29	Female	Serum	June 2, 2022	Negative
6	BEN-22-MPOX 003	29	Female	Skin swab of the lesions	June 2, 2022	Positive (Ct: 23.44)
7	BEN-22-MPOX 004	15	Male	Serum	June 5, 2022	Negative
8	BEN-22-MPOX 004	15	Male	Skin swab of the lesions	June 5, 2022	Positive (Ct: 34.00)
9	BEN-22-MPOX 005	22	Male	Serum	June 6, 2022	Negative
10	BEN-22-MPOX 005	22	Male	Skin swab of the lesions	June 6, 2022	Negative
11	BEN-22-MPOX 006	6	Female	Serum	June 7, 2022	Negative
12	BEN-22-MPOX 006	6	Female	Skin swab of the lesions	June 7, 2022	Negative
